# Celebrating five years of *BJR*|*Open*

**DOI:** 10.1093/bjro/tzae009

**Published:** 2024-05-10

**Authors:** Katja Pinker, Habib Zaidi

**Affiliations:** Memorial Sloan Kettering Cancer Center, New York, NY, United States; Columbia University Irving Medical Center, New York, NY, United States; Geneva University Hospital, Geneva, Switzerland

**As *BJR|Open* turns five, the Editors-in-Chief reflect on the journal’s mission, achievements, and ambitions for the years to come.**

Five years ago, in 2018, a new journal, *BJR*|*Open*, was introduced to provide an open access, online-only, international research journal published by the British Institute of Radiology (BIR) to add to and further strengthen the established *BJR* family of journals.

While *BJR*|*Open* mirrors the scope of its sister journals *BJR, BJR*|*Artificial Intelligence* and *BJR*|*Case Reports*, covering clinical and technical aspects of medical imaging, radiotherapy, radiation oncology, medical physics, radiobiology, and the underpinning sciences, it notably is a multidisciplinary journal that offers a wholly open access option to the medical imaging and wider research community. *BJR*|*Open* strives not only to be essential reading for radiologists, medical physicists, radiobiologists, radiation oncologists, radiotherapists, radiographers, and any healthcare professionals or researchers with an interest in medical imaging alike, but as a fully open access journal aims to reach any interested reader in the world with an internet connection.

The first volume of *BJR*|*Open* was published in 2019 and in 2024 the journal is now in its sixth volume. The journal has received submissions from all around the world and across all specialties. Significant milestones in the young history of *BJR*|*Open* were reached with being indexed in PubMed in 2021 followed by inclusion in the Web of Science Core Collection, in the Emerging Sources Citation Index, in 2024, meaning that *BJR*|*Open* will receive its first Journal Impact Factor (JIF) soon. This shows the quality of content published in the journal and how it is valued by its readers. The journal features original Research Articles, Reviews, Pictorial Reviews, Practice and Policy papers, Opinion pieces and Letters to the Editor. *BJR*|*Open* also considers papers transferred from sister journal *BJR*, which highlights the strong bonds between the BIR journal family.

Five years later, we, as Editors-in-Chief, are truly proud of what *BJR*|*Open* has become and we would like to highlight some recent exciting developments within the journal.

*BJR*|*Open* now features themed content in order to highlight current hot topics and emerging areas of research. Two Special collections were launched in 2023; one on “Evolving role of AI in radiation oncology”, guest edited by Professor Issam El Naqa, Professor Weigang Hu, Dr Sarah Mattonen and Professor Esther Troost, and another on the “Advances in cancer imaging and technology”, guest edited by Professor Regina Beets-Tan, Dr Zuhir Elkarghali, Dr Sharyn Katz, and Professor Haibin Shi. The aim of these special collections is to grow over time and become a go-to resource for high quality research on a particular topic. We are looking forward to more special collections in the journal’s future, with one on the global impact of radiology planned to launch during 2024.

*BJR*|*Open* also has a social media presence and readers are encouraged to follow @BJR_Radiology on X (previously known as Twitter), which has over 12 000 followers, and like, repost and quote posts about *BJR*|*Open* content. As a young journal, we are grateful for strong support from the community in helping to promote the journal.

In addition, articles published in *BJR*|*Open* are regularly featured in BIR podcasts. The BIR publishes regular podcasts that are all freely available to listen to and download on the BIR website. Podcasts act as another avenue of discoverability and promotion for the journal, and listeners can hear journal editors, authors, opinion leaders, and independent experts discuss selected articles from *BJR*|*Open* and the other BIR journals, as well as timely topics within the field of medical imaging.

The scientific quality of a peer-reviewed journal relies on many factors, among which its aptitude to entice prominent scientists and distinguished colleagues who find the journal a commendable venue to publish their research findings. Under the stewardship of the editors and BIR staff team, and with the substantial assistance of the expertise of its busy international reviewers and Editorial Board members, one of the objectives for the journal is to continue to nurture in stature and to ensure a reasonably fast turnaround time without loss of quality. Among our future objectives is also to enhance content and presentation and provide a better balance between contributions from clinical research and basic science.

It has been an extraordinary privilege and pleasure to help guide this journal in its first five years and we are looking forward to many more. We whole-heartedly thank all the authors who have trusted us with their work and embarked together with us on this journal’s journey. Without our outstanding Senior Editors, our WPN Editorial Advisory Board and reviewers who generously dedicate their expertise and time and provide invaluable and constructive feedback to authors to help them improve their manuscripts, this endeavour would not be possible. See Table 1 for a list of Senior Editors.

**Table 1.** BJR|*Open* Senior Editors.

*BJR*|*Open* Senior Editors	
**Diagnostic imaging**	
Breast	Dr Federica Pediconi Sapienza University of Rome, Rome, Italy
Cardiac	Dr Tessa Cook University of Pennsylvania, Philadelphia, PA, United States
Gastrointestinal	Professor Daniele Regge Candiolo Cancer Institute—IRCCS, Turin, Italy
Genitourinary	Dr Alberto Vargas NYU Langone Health, New York, NY, United States
Head & Neck and Neuroradiology	Dr Sotirios Bisdas UCL Institute of Neurology, London, United Kingdom
Interventional radiology	Dr Florian Wolf Medical University of Vienna, Vienna, Austria
Musculoskeletal	Professor Hema Choudur McMaster University, Hamilton, Ontario, Canada
Nuclear Medicine & Molecular Imaging	Dr Marius Mayerhoefer NYU Langone Health, New York, NY, United States
Obstetrics & Gynaecology	Professor Isabelle Thomassin-Naggara Sorbonne University, Paris, France
Paediatric Radiology	Dr Susan Shelmerdine Great Ormond Street Hospital for Children NHS Foundation Trust, London, United Kingdom
Respiratory/Chest	Dr Helmut Prosch Medical University of Vienna, Vienna, Austria
**Physics and technology**	
Artificial Intelligence and Machine Learning	Dr Jonas Teuwen Netherlands Cancer Institute, Amsterdam, Netherlands
Diagnostic Physics	Dr Sunitha Thakur Memorial Sloan Kettering Cancer Center, New York, NY, United States
Radiotherapy Physics	Dr David Thomas University of Colorado School of Medicine, Aurora, CO, United States
**Radiotherapy and oncology**	
Radiotherapy and Oncology	Dr Luca Boldrini Fondazione Policlinico Universitario Agostino Gemelli IRCCS, Rome, Italy
**Radiobiology**	
Radiobiology	Professor Guangming Zhou Soochow University, Suzhou, China

The BIR has provided support, and the competent and dedicated *BJR*|*Open* Publications staff team, including Miranda Ashby-Wood, Tami Potten, and Maryam Miah, have guided our journal’s operations.

As *BJR*|*Open* moves into its sixth year of publication, and its sixth volume, we as Editors-in-Chief want to extend our heartfelt thanks to all who have helped support the journal’s successes to date and hope for their continued support. Our journal provides a reliable and safe outlet for open access publication, where authors and readers can feel reassured by our robust and rigorous peer review process. The future of *BJR*|*Open* looks bright and healthy as it strives to offer to clinicians and researchers a model showcase for high quality research manuscripts and a recognized medium for new research challenges.

